# Impact of anaemia severity on functional outcome in patients with cerebral venous thrombosis: a DOAC-CVT substudy

**DOI:** 10.1093/esj/aakag006

**Published:** 2026-02-27

**Authors:** Nada M Sobih, Jelle Vellema, Anita van de Munckhof, Mayte Sanchez van Kammen, Xunming Ji, Ido van den Wijngaard, Jeremy Molad, Marialuisa Zedde, Y Muralidhar Reddy, Mohammad Wasay, Antonio Arauz, Mirjam Heldner, Nesrin Ergin, Miguel A Barboza, Diana Aguiar de Sousa, Katarina Jood, Jukka Putaala, Turgut Tatlisumak, Jose M Ferro, Jonathan M Coutinho

**Affiliations:** Department of Neurology, Amsterdam UMC, location University of Amsterdam, Amsterdam, The Netherlands; Department of Neurology, Amsterdam UMC, location University of Amsterdam, Amsterdam, The Netherlands; Department of Neurology, Amsterdam UMC, location University of Amsterdam, Amsterdam, The Netherlands; Department of Neurology, Amsterdam UMC, location University of Amsterdam, Amsterdam, The Netherlands; Department of Neurology, Xuanwu Hospital, Capital Medical University, Beijing, China; Department of Neurology, Haaglanden Medical Center, The Hague, The Netherlands; Department of Neurology, Tel Aviv Sourasky Medical center, Tel Aviv, Israel; Department of neurology, Azienda Unita Sanitaria Locale di Reggio Emilia, Reggio Emilia, Italy; CARE Institute of Neurological Sciences, CARE Hospital, Banjara Hills, Hyderabad, India; Department of Neurology, Aga Khan University, Karachi, Pakistan; Department of Neurology, Instituto Nacional de Neurologia y Neurocirugía Manuel Velasco Suarez, Mexico City, Mexico; Department of Neurology, Inselspital, University Hospital and University of Bern, Bern, Switzerland; Department of Neurology, Medical Faculty, Pamukkale University, Denizli, Turkey; Neurosciences Department, Hospital Dr. Rafael A. Calderón Guardia, San José, Costa Rica; Department of Neurosciences, Stroke Center, Centro Hospitalar Universitário de Lisboa Central – ULS São José, CEEM and Institute of Anatomy, Faculdade de Medicina da Universidade de Lisboa, Gulbenkian Institute of Molecular Medicine, Lisbon, Portugal; Department of Neurology, Sahlgrenska University Hospital, Gothenburg, Sweden; Department of Clinical Neuroscience, Institute of Neuroscience and Physiology, Sahlgrenska Academy at University of Gothenburg, Gothenburg, Sweden; Department of Neurology, Helsinki University Hospital and University of Helsinki, Helsinki, Finland; Department of Neurology, Sahlgrenska University Hospital, Gothenburg, Sweden; Department of Clinical Neuroscience, Institute of Neuroscience and Physiology, Sahlgrenska Academy at University of Gothenburg, Gothenburg, Sweden; Hospital da Luz, University of Lisbon, Lisbon, Portugal; Department of Neurology, Amsterdam UMC, location University of Amsterdam, Amsterdam, The Netherlands

**Keywords:** cerebral venous thrombosis, cerebral venous sinus thrombosis, functional outcome, prognosis, anaemia, haemoglobin, disability

## Abstract

**Introduction:**

Anaemia is an established risk factor for poor outcome in intracerebral haemorrhage and ischaemic stroke. We examined whether anaemia predicts poor outcome in cerebral venous thrombosis (CVT).

**Patients and methods:**

We used data of the DOAC-CVT study, which was an international, prospective observational cohort study in adult patients with CVT that ran from January 2021 to January 2024. Anaemia at admission was defined according to World Health Organization criteria. Poor outcome was defined as modified Rankin Scale (mRS) 3–6 at 6-months. Binary logistic regression, adjusted for age, recent delivery/puerperium, income country, cancer and intracranial haemorrhage, was applied.

**Results:**

Of 619 patients in DOAC-CVT, 583 patients were included, of whom 157 (27%) had anaemia. Compared to patients without anaemia, patients with anaemia were slightly younger (median age 40 vs. 42 years), more often female (76% vs. 59%), from middle income countries (36% vs. 21%), more often had intracranial haemorrhage (48% vs. 32%) and cancer (5% vs. 2%). Anaemia was associated with poor functional outcome (mRS 3–6, 10% vs. 5%, aOR: 2.20, 95% Cl, 1.01–4.81), but not with mortality (3% vs. 1%, aOR: 3.54, 95% Cl, 0.68–18.31). When stratified by severity, moderate to severe anaemia was associated with poor functional outcome (aOR 2.88, 95% Cl, 1.14–7.38), but mild anaemia was not (aOR 1.64, 95% Cl, 0.60–4.55).

**Discussion and conclusion:**

Anaemia at admission, especially moderate to severe, is a predictor for poor functional outcome in patients with CVT, highlighting the need for further studies on potential interventions.

## Graphical Abstract

**Graphical Abstract:**
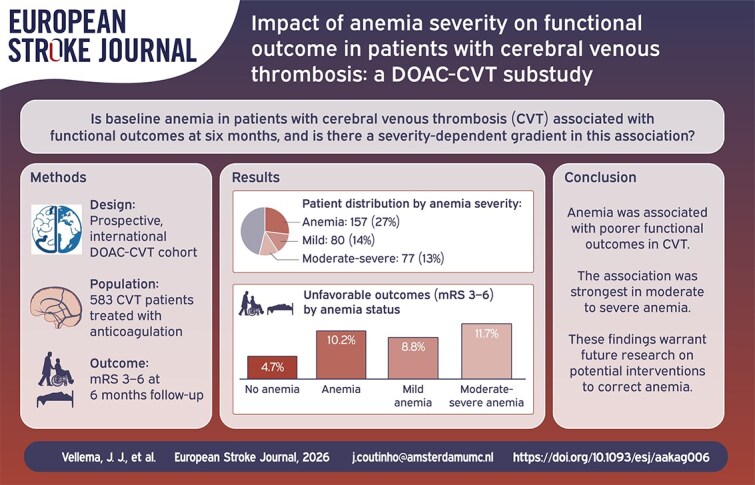

## Introduction

Cerebral venous thrombosis (CVT) is an uncommon cerebrovascular disease, affecting approximately 1–2 per 100 000 individuals annually.^1^ Although the prognosis of CVT is favourable in many patients, a minority experiences persistent functional impairment or fatal outcomes.^2^ Several clinical variables that predict poor outcome have been previously identified in patients with CVT, including intracranial haemorrhage, thrombosis of the deep venous system, decreased consciousness, older age, coma and cancer.^3–6^

Anaemia is present in 7%–27% of patients with CVT at diagnosis and has previously been identified as an independent risk factor for disease occurrence.^2,7,8^ More recently, anaemia has also been recognized as a predictor of poor outcome in other cerebrovascular diseases, such as ischaemic and haemorrhagic stroke.^9–11^ The impact of anaemia on clinical outcomes is thought to be related to impaired oxygen delivery, heightened inflammatory responses, altered blood viscosity and decreased physiological reserve.^12–17^ Similar pathophysiological mechanisms might underlie an association between anaemia and poor outcomes in CVT.

To date, only 2 studies have investigated the relationship between anaemia and outcomes after CVT.^18,19^ In both studies, anaemia was associated with worse clinical outcomes, with the strongest associations seen in patients with moderate to severe anaemia. Both studies were predominantly retrospective in nature and had limited sample size. The aim of the current study was to investigate whether anaemia at baseline is associated with poor functional outcomes in patients with CVT, using data from a large international prospective cohort study.

## Patients and methods

### Study design and population

We performed a post hoc analysis of the DOAC-CVT study. DOACT-CVT was a prospective, international, observational cohort designed to investigate the effectiveness and safety of direct oral anticoagulants (DOACs) compared to vitamin K antagonists (VKAs) in adult patients with CVT. The study protocol and primary results of the study have been published.^20,21^ The study was conducted in 65 hospitals, across 23 countries on 5 continents. Patients could be included if they were 18 years or older, if CVT was radiologically confirmed, and if oral anticoagulant therapy (either DOAC or VKA) had been started within 30 days of diagnosis. Exclusion criteria were the use of anticoagulation before CVT diagnosis, pregnancy or breast feeding and a contraindication to DOACs.^21^ For this substudy, we additionally excluded patients for whom baseline haemoglobin or follow-up data on functional recovery were missing. Detailed baseline data were collected and patients were followed up at 3 and 6 months after diagnosis. All data were entered in an electronic data capture system (Castor EDC). All participants or their immediate family members provided written informed consent. The study was conducted in accordance with the Declaration of Helsinki and Good Clinical Practice guidelines, and was approved by relevant ethics committees and regulatory authorities in each participating country.

### Haemoglobin assessment, anaemia classification and outcomes

Haemoglobin concentration was measured at hospital admission as part of routine clinical care. Values were converted to millimoles per litre (mmol/L) when necessary. Anaemia was defined according to World Health Organization criteria as a haemoglobin level of <8.1 mmol/L (<13.0 g/dL) in men and <7.5 mmol/L (<12.0 g/dL) in women. For subgroup analyses, anaemia severity was classified as mild (6.8–8.0 mmol/L [11–12.9 g/dL] in men; 6.8–7.4 mmol/L [11–11.9 g/dL] in women) or moderate to severe (<6.8 mmol/L [<11 g/dL] for both men and women).^22^

The primary outcome was poor functional outcome at 6-months follow-up, assessed using the modified Rankin Scale (mRS). A score of 0 on the mRS indicates no symptoms; 1, no significant disability; 2, slight disability; 3, moderate disability; 4, moderately severe disability; 5, severe disability requiring constant care; and 6, death.^23^ Outcomes were dichotomized into favourable (mRS 0–2) and unfavourable (mRS 3–6).

### Statistical analysis

Baseline characteristics were compared between anaemic and non-anaemic patients. Categorical variables are reported as frequencies (%) and compared using Chi-squared or Fisher’s exact test, as appropriate. Continuous variables are presented as means with standard deviation or median (interquartile range, IQR), depending on distribution, and were compared using *t*-tests or Mann–Whitney U tests.

To assess the relationship between anaemia and unfavourable outcome, three binary logistic regression models were used: The first model assessed the impact of anaemia as a dichotomous variable (anaemic vs. non-anaemic) on the likelihood of an unfavourable outcome (mRS 3–6). The second model investigated the association between haemoglobin levels as a continuous variable and unfavourable outcomes (mRS 3–6). The third model examined the impact of anaemia as a dichotomous variable on the 6-month mortality rate.

For further analysis, two multivariate ordinal regression models were conducted to evaluate the impact of anaemia across the entire spectrum of mRS scores (0–6). Two separate models were used assessing haemoglobin as a continuous variable and anaemia status as a categorical variable (anaemic vs. non-anaemic).

We conducted two sensitivity analyses. In the first, patients with cancer were excluded. Cancer was defined as active malignancy requiring treatment or cancer diagnosed within 6 months prior to the CVT event. In the second sensitivity analysis, to account for baseline functional status, patients with a premorbid mRS score greater than 2 were excluded. In addition, subgroup analyses were conducted according to anaemia severity (mild and moderate to severe), gender and clinical subgroups typically associated with anaemia, including young women of reproductive age (18–50), older patients (>65 years) and patients with cancer or autoimmune diseases, using binary logistic regression.

All models were adjusted for age, recent delivery, country income level, intracranial haemorrhage and cancer. In the subgroup analyses, the variables cancer, recent delivery and age were omitted when they overlapped with the defining characteristic of the subgroup, such as in analyses of cancer patients, young women, men and older patients.^2,24^ Missing data were handled by complete-case analysis. Odds ratios (ORs) with corresponding 95% CIs were reported, and a 2-sided *P*-value <.05 was considered statistically significant. Analyses were performed using IBM SPSS Statistics version 24 (IBM Corp., Armonk, NY, USA).

## Results

**Figure 1:**
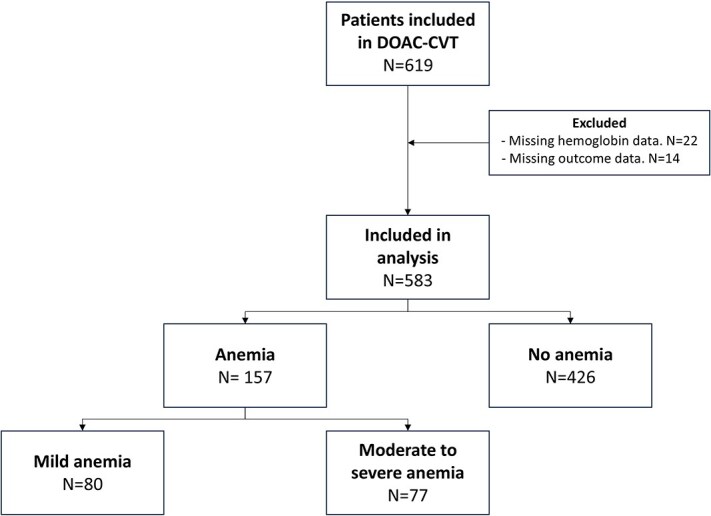
Flowchart of patient inclusion. This figure illustrates the inclusion and exclusion process for the study cohort. It further categorizes patients into anaemic and non-anaemic groups, with anaemia severity further stratified into mild and moderate to severe categories. Abbreviation: CVT = cerebral venous thrombosis.

Between January 2021 and January 2024, 619 patients with CVT were enrolled in the DOAC-CVT study. Of these, 36 patients were excluded because of missing baseline haemoglobin levels (22) or follow-up data (14), leaving 583 patients for analysis. Of these, 157 (26.9%) had anaemia at baseline and 426 (73.1%) did not. Among those with anaemia, 80 patients (13.7%) had mild anaemia and 77 patients (13.2%) had moderate to severe anaemia (Figure 1). In total, 332 patients (57.1%) were from high-income countries and 251 patients (43.1%) were from middle-income countries.

Median haemoglobin level was 6.8 mmol/L (IQR 5.5–7.3) in the anaemic group and 8.8 mmol/L (IQR 8.3–9.5) in the non-anaemic group. Anaemic patients were younger (median age 40 vs. 42 years, *P* = .01), more often female (76.4% vs. 58.7%, *P* < .01), and more frequently from middle-income countries (35.5% vs. 20.5%; *P* < .001). Cancer (4.5% vs. 1.9%, *P* = .14) and auto-immune disease (10.2 vs 4.2%, *P* < .01) were more prevalent in patients with anaemia. Anaemic patients presented more frequently with focal neurological deficits (53.5% vs. 35.9%, *P* < .01), lower Glasgow Coma Scale scores (median: 14 [IQR: 13–15] vs. 15 [IQR: 15–15], *P* < .01), and intracranial haemorrhage (48.4% vs. 31.5%, *P* < .01; Table 1).

**Table 1:** Baseline table of the anaemia group compared to the no anaemia group.

	**No anaemia** **(*n* = 426)**	**Anaemia** **(*n* = 157)**	***P*-value**
**Patient characteristics, *n/N* (%)**			
Age, median (IQR), years	42 (29–53)	40 (27–48)	.011
Female sex	250/426 (58.7)	120/157 (76.4)	<.001
Country of inclusion’s income group^1^			
High-income country	264/332 (79.5)	68/332 (20.5)	<.001
Middle-income country	162/251 (64.5)	89/251 (35.5)	
Risk factors			
Oral contraceptives^2^	116/250 (46.4)	35/120 (29.2)	.243
Recent delivery, puerperium^3^	11/250 (4.4)	9/120 (7.5)	.217
Other prothrombotic medication	28/426 (6.6)	6/157 (3.8)	.238
Head or neck infection^4^	29/426 (6.8)	17/157 (10.8)	.120
CNS infection	5/426 (1.2)	4/157 (2.5)	.260
Cancer^5^	8/426 (1.9)	7/157 (4.5)	.135
Autoimmune disease	18/426 (4.2)	16/157 (10.2)	.009
Known APS or presence of antiphospholipid antibodies at baseline	36/426 (8.5)	12/157 (7.6)	.866
Previous VTE	33/426 (7.7)	8/157 (5.1)	.283
Previous major bleeding^6^	6/426 (1.4)	7/157 (4.5)	.050
No risk factor identified	155/426 (36.4)	33/157 (21.0)	<.001
Pre-morbid mRS 3–6	12/426 (2.8)	10/157 (6.4)	.043
**Presenting symptoms, *n/N* (%)**			
Time from symptom onset to diagnosis, median (IQR), days	4 (1–11)	3 (1–10.75)	.577
Headache	383/426 (89.9)	140/157 (89.2)	.878
Focal neurologic deficits	153/426 (35.9)	84/157 (53.5)	<.001
Seizure	117/426 (27.5)	46/157 (29.3)	.678
GCS, median (IQR)	15 (15–15)	15 (13–15)	<.001
Coma^7^	10/426 (2.3)	8/157 (5.1)	.106
**Baseline radiological characteristics, *n/N* (%)**			
Location thrombosis			
Superior sagittal sinus	214/426 (50.2)	67/157 (42.7)	.113
Straight sinus	70/426 (16.4)	19/157 (12.1)	.243
Deep venous system	31/426 (7.3)	10/157 (6.4)	.722
Cortical vein	98/426 (23.0)	32/157 (20.4)	.505
Number of thrombosed sinuses, median (IQR)	3 (2–4)	3 (2–4)	.340
Non-haemorrhagic lesion	94/426 (22.1)	43/157 (27.4)	.187
Intracranial haemorrhage	134/426 (31.5)	76/157 (48.4)	<.001
**Baseline laboratory parameters**			
White blood cell, 10^9^/L	9.0 (7.0–11.2)	9.7 (7.0–13.0)	.125
Platelet count, 10^9^/L	254.5 (209.8–311.2)	302.0 (231.3–399.5)	<.001
C-reactive protein, mg/L	5.81 (2.00–16.70)	9.21 (3.00–41.10)	.002
Renal function (eGFR) mL/min/1.73 m^2^	94.0 (81.0–112.0)	100.0 (85.5–120.1)	.057
Creatinine (mg/dL)	1.17 (0.80–1.20)	0.85 (0.61–0.85)	<.001
**Initial treatment data, n/N (%)**			
Lead-in parenteral anticoagulants	394/426 (92.5)	146/157 (93.0)	.862
Endovascular treatment	21/426 (4.9)	7/157 (4.5)	.834
Decompressive neurosurgery	4/426 (0.9)	8/157 (5.1)	.004
Intensive care unit admission	67/426 (15.7)	37/157 (23.6)	.038

This table presents the baseline characteristics of the anaemic and non-anaemic groups. Abbreviations: CNS = central nervous system; eGFR = estimated glomerular filtration rate; GCS = Glasgow Coma Scale; ICU = intensive Care Unit; IQR = interquartile range; LMWH = low molecular weight heparin; mRS = modified Rankin Scale; VTE = venous thromboembolis.

^1^According to the criteria of the World Bank.

^2^Percentage of women.

^3^Within 12 weeks of symptom onset.

^4^Within 1 week.

^5^Diagnosis within 6 months of symptom onset or currently undergoing active treatment.

^6^According to criteria of International Society on Thrombosis and Haemostasis.

^7^Glasgow Coma Scale score ≤ 8.

**Figure 2:**
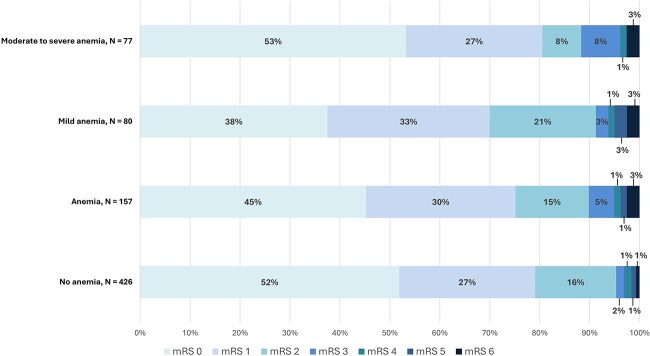
Stacked distribution of modified Rankin scale outcomes at 6 months across anaemia categories. This figure illustrates the stacked bar chart of mRS scores at 6-moths follow-up for each anaemia category (no anaemia, anaemia, mild anaemia, moderate-to-severe anaemia). For each mRS score, the corresponding percentage is indicated within the bars. Notably, no patients in the moderate to severe anaemia group had a mRS score of 5. Abbreviation: mRS = modified Rankin scale.

After 6-months follow-up, 547 patients (93.8%) had a favourable functional outcome (mRS 0–2) and 36 patients (6.2%) a unfavourable outcome (mRS 3–6). Both unfavourable functional outcome (10.2% vs. 4.7%, *P* = .01) and mortality (2.5% vs. 0.7%, *P* = .07) were more common among patients with anaemia than those without anaemia (Figure 2). After adjustment, the presence of anaemia was associated with an increased risk of unfavourable functional outcome (mRS 3–6; aOR: 2.20, 95% CI, 1.01–4.81; Table 2). When anaemia was modelled as a continuous variable, lower haemoglobin levels were also associated with unfavourable outcomes (aOR per one mmol/L increase in haemoglobin: 0.75, 95% CI, 0.61–0.93). Ordinal regression analysis indicated that anaemia was statistically non-significant associated with a shift toward worse functional outcomes across the full mRS spectrum (aOR: 1.43, 95% CI, 0.99–2.05). This finding was consistent when haemoglobin was analysed as continuous variable (aOR per one mmol/L increase in haemoglobin: 0.92; 95% CI, 0.83–1.02). In addition, mortality was nominally higher among anaemic patients but this difference was not statistically significant (aOR: 3.54, 95% CI, 0.68–18.31; Table 2).

**Table 2:** Association of anaemia with poor functional outcomes (mRS 3–6) and mortality.

	**No. of patients *n/N* (%)**		**Unadjusted OR, (95% Cl)**	**Adjusted OR, (95% Cl)**
	**No anaemia**	**Anaemia**		
**mRS 3–6**	20/426 (4.7%)	16/157 (10.2%)	2.30 (1.16–4.57)	2.20 (1.01–4.81)
**Mortality**	3/426 (0.7%)	4/157 (2.5%)	3.67 (0.82–16.66)	3.54 (0.68–18.31)

Outcomes of the binary logistic regression analysis. Adjusted for age, recent delivery, cancer, income group and intracranial haemorrhage. Abbreviations: mRS = modified Rankin Scale; OR = Odds Ratio.

The prevalence of unfavourable functional outcome at 6-months increased with anaemia severity and was 4.7% in patients without anaemia, 8.8% in those with mild anaemia and 11.7% in those with moderate to severe anaemia (Figure 2). Moderate to severe anaemia was associated with mRS 3–6 (aOR: 2.88, 95% CI, 1.14–7.38; *P* = .03), whereas mild anaemia was not (aOR: 1.64, 95% CI, 0.60–4.55; *P* = .35; Table 3). The presence of anaemia, irrespective of severity, showed a statistically significant association with unfavourable functional outcome (mRS 3–6) in women (aOR: 3.61, 95% CI, 1.04–8.82; *P* = .03) but not in men (aOR: 2.13; 95% CI, 0.54–8.30; *P* = .28). In the subgroups of patients with cancer or autoimmune disease (aOR: 1.16; 95% CI, 0.19–7.07; *P* = .88), and older patients (aOR: 1.09; 95% CI, 0.23–5.19; *P* = .91), anaemia showed no significant association with unfavourable functional outcome. Among young women, the presence of anaemia was associated with a statistically non-significant trend toward unfavourable functional outcomes (aOR 4.28; 95% CI, 0.95–19.18; *P* = .06).

**Table 3:** Association of anaemia severity with poor functional outcome (mRS 3–6).

	**No. of Patients *n/N* (%)**		**Unadjusted OR, (95% Cl)**	**Adjusted OR, (95% Cl)**
	**mRS 0–2**	**mRS 3–6**		
**Haemoglobin level**				
Mild anaemia	73/547 (13.3%)	7/36 (19.4%)	1.95 (0.80–4.77)	1.64 (0.60–4.55)
Moderate–severe anaemia	68/547 (12.4%)	9/36 (25.0%)	2.69 (1.17–6.15)	2.88 (1.14–7.38)

Outcomes of the binary logistic regression analysis stratified by sex. Adjusted for age, cancer, income group, recent delivery and intracranial haemorrhage. Abbreviations: mRS = modified Rankin Scale; OR = odds ratio.

In a sensitivity analysis excluding patients with cancer (*n* = 15), anaemia remained associated with unfavourable outcome (aOR: 2.34; 95% CI, 1.02–5.39; *P* = .045). In the sensitivity analysis excluding patients with a premorbid mRS score higher than 2 (*n* = 22) and with haemoglobin modelled as continuous variable, the association with unfavourable outcome remained significant (aOR per one mmol/L increase in haemoglobin: 0.76, 95% CI, 0.60–0.97). When modelled as a dichotomous variable, anaemia was associated with higher odds of unfavourable functional outcome (aOR 1.86; 95% CI, 0.75–4.62), though this association was no longer statistically significant.

## Discussion

In this post hoc analysis of the DOAC-CVT study, anaemia was present at baseline in approximately one in four patients with CVT. The presence of anaemia was independently associated with poor functional outcome (mRS 3–6) at follow-up. The strongest prognostic effect was present in moderate to severe anaemia.

The rate of anaemia is consistent with previous studies, which reported rates ranging from 22% to 27%.^7,25^ The strength of association between the dichotomized anaemia and poor outcome (aOR 2.2) is also in line with findings from Silvis et al. and Kai Liu et al. (aORs 2.4 and 3.0, respectively) (14, 15). In contrast, mortality rates varied substantially. We observed a 6-month mortality of 3% among anaemic patients, compared to 11% and 27% in the studies by Silvis and Kai Liu. Several factors may explain this discrepancy in mortality. In the study by Silvis et al., both coma and cancer were more prevalent (11% and 17%, respectively) than in the current study (5% and 5%), both of which are established predictors of mortality in CVT. In the study by Kai Liu et al., the rate of coma was also considerably higher than in our study. This difference in clinical severity may be explained by the fact that some of the more severe cases were not included in DOAC-CVT because of early death (ie, prior to start of oral anticoagulation) or because patients were not started on oral anticoagulation within 30 days, for instance because of cancer or difficulty swallowing.

The majority of patients with anaemia were from middle-income countries, likely reflecting the higher prevalence of anaemia in these regions due to nutritional, socioeconomic and public health factors.^26^ Notably, after adjusting for country income level, anaemia remained independently associated with poorer functional outcomes. This suggests that, while country’s income status may influence the incidence of CVT with concomitant anaemia, it does not fully explain the observed association between anaemia and worse CVT outcomes.

The impact of anaemia on functional outcome followed a severity gradient, with moderate to severe anaemia exhibiting a stronger relation than in mild anaemia. This supports the hypothesis that cerebral oxygen delivery becomes critically impaired below a hemodynamic threshold, whereas mild anaemia may be offset by compensatory mechanisms such as increased cardiac output and oxygen extraction.^27^

The effect of anaemia appeared to be more pronounced in women, although this could be due to a reduced statistical power among men. Silvis et al. reported an increased risk in both sexes but did not explore potential sex-specific modifiers.^18^ In related conditions such as acute ischaemic stroke, the association between worse outcomes and female sex appears to be partly attributable to lower haemoglobin levels in women.^28^ Stratification by clinical subgroups typically associated with anaemia showed no significant association with poorer outcomes among patients with cancer, autoimmune disease or older age, whereas a non-significant trend towards worse outcomes was observed in young women. These findings should be interpreted with caution, as small sample sizes likely limited statistical power despite considerable numerical differences in the cancer/autoimmune disease and older patient groups. Furthermore, interpreting outcomes of patients with cancer or autoimmune disease is challenging, as this subgroup includes highly heterogeneous conditions with widely varying prognoses. Further studies are therefore warranted to determine whether anaemia exerts sex-specific effects and whether its impact is significant in patients with cancer or autoimmune disease, as well as to elucidate the mechanisms underlying these potential differences.

Although several biological mechanisms have been proposed through which anaemia may exacerbate neurological injury—such as impaired cerebral oxygen delivery, disrupted autoregulation, endothelial dysfunction and increased inflammation—our observational design precludes determination of their causal involvement.^12–17^ Alternatively, anaemia may reflect underlying frailty or chronic disease, including systemic inflammation, malnutrition, occult malignancy or other comorbid conditions, and thus function as a biological marker rather than a direct mediator of unfavourable outcome. The higher proportion of severe clinical features among CVT patients with anaemia, such as intracerebral haemorrhage, focal neurological deficits, decompressive surgery and ICU admission, could support this hypothesis. However, these features could also be downstream consequences of anaemia itself. In this context, the coexistence of CVT and anaemia may represent a “second-hit,” where anaemia, by impairing oxygen transport and exacerbating cerebral vulnerability, predisposes patients to more severe neurological injury. This, in turn, may lead to worse outcomes, such as intracerebral haemorrhage and neurological deficits, and a greater need for intensive interventions.

It is unknown whether correcting anaemia improves the outcome of patients with CVT. A recent randomized controlled trial did demonstrate that, among patients with acute brain injury and anaemia, a liberal red blood cell transfusion threshold was associated with improved clinical outcomes compared to a restrictive transfusion strategy.^29^ However, extrapolation to CVT is not straightforward. The pathophysiological profile of CVT, characterized by venous congestion and potentially elevated intracranial pressure, may increase patients’ susceptibility to transfusion-related complications, including hyperviscosity and thrombotic propagation.^30^ Nevertheless, the more pronounced association between moderate to severe anaemia and poor functional outcome may suggest that this subgroup could benefit from targeted correction strategies, and this should be investigated in future studies.

Our study has several limitations. Although the DOAC-CVT study was prospectively designed, anaemia was not a predefined primary outcome, limiting causal inferences. Still the rate of missing data of haemoglobin levels was low (4%). However, additional details, including cause of anaemia and additional laboratory values such as MCV, and data on anaemia interventions were not collected. Second, although we adjusted for several potential confounders, residual confounding cannot be excluded, either due to baseline imbalances or unmeasured variables that may partly explain the observed association between anaemia and poor outcomes. Third, the absence of patients included from low-income countries, where anaemia is generally more common, and the exclusion of pregnant women limit generalizability of the findings to these populations. Fourth, we did not have information on the underlying causes of anaemia, and therefore were unable to perform stratified analyses by anaemia type. Fifth, we lacked information on longitudinal changes in haemoglobin levels or on anaemia correction during follow-up, both of which may have influenced clinical outcome. Sixth, because cancer is not systematically screened for in patients with CVT, underreporting of malignancy is possible. Finally, the described population does not represent the entire spectrum of CVT patients, as it is limited to those who progressed to oral anticoagulation, potentially resulting in the preferential inclusion of less severe cases.

In conclusion, we found that anaemia is associated with poor functional outcome in patients with CVT. The association was more pronounced in patients with moderate to severe anaemia. Further studies are needed to determine whether correcting anaemia at baseline improves the outcome of patients with CVT.

## Supplementary Material

aakag006_Supplemental_material

## Data Availability

De-identified participant data will be available upon reasonable request, in compliance with the General Data Protection Regulation. All data requests must be reviewed and approved by the DOAC-CVT executive committee. Interested researchers can submit their proposals to j.coutinho@amsterdamumc.nl.
