# Supplementary material for: Impact of anaemia severity on functional outcome in patients with cerebral venous thrombosis: a DOAC-CVT substudy
Source: Eur Stroke J. 2026 Feb 27;11(2):aakag006. doi: 10.1093/esj/aakag006 (PMC12947706; doi:10.1093/esj/aakag006)
Supplement: aakag006_Supplemental_material [file aakag006_supplemental_material.docx]

**Supplemental material**

**Table 4. Association of anemia with poor functional outcome (mRS 3–6) stratified in subgroups**

|  | **No. of patients n/N (%)** | | **Unadjusted OR,  (95% Cl)** | **Adjusted OR,  (95% Cl)** |
| --- | --- | --- | --- | --- |
|  | No anemia mRS 3-6 | Anemia mRS 3-6 |  |  |
| **Gender** |  |  |  |  |
| Male participants | 8/176 (4.5%) | 5/37 (13.5%) | 3.28 (1.01-10.67) | 2.13 (0.54-8.30)a |
| Female participants | 12/250 (4.8%) | 11/120 (9.1%) | 2.00 (0.86-4.68) | 3.61 (1.04-8.82)b |
| **Classification analysis** |  |  |  |  |
| Cancer and auto-immune disease | 4/26 (15.4%) | 5/23 (21.7%) | 1.53 (0.36-6.56) | 1.16 (0.19-7.07)c |
| Young women (18-50 years) | 3/169 (1.8%) | 7/99 (7.1%) | 4.21 (1.06-16.67) | 4.28 (0.95-19.18)a |
| Older patients (>65 years) | 9/47 (19.1%) | 4/13 (30.8%) | 1.89 (0.47-7.49) | 1.09 (0.23-5.19)d |

a Adjusted for age, cancer, income group and intracranial hemorrhage; b Adjusted for age, recent delivery, cancer, income group and intracranial hemorrhage. c Adjusted for age, income group, recent delivery and intracranial hemorrhage. d Adjusted for cancer, income group and intracranial hemorrhage. mRS = Modified Rankin Scale; OR = Odds Ratio; CI = Confidence Interval.
